# Evidence of Multiple Disease Resistance (MDR) and Implication of Meta-Analysis in Marker Assisted Selection

**DOI:** 10.1371/journal.pone.0068150

**Published:** 2013-07-10

**Authors:** Farhan Ali, Qingchun Pan, Genshen Chen, Kashif Rafiq Zahid, Jianbing Yan

**Affiliations:** 1 National Key Laboratory of Crop Genetic Improvement, Huazhong Agricultural University, Wuhan, China; 2 Cereal Crops Research Institute (CCRI) Pirsabak Nowshera, Khyber Pakhtunkhwa, Pakistan; 3 National Maize Improvement Center of China, China Agricultural University, Beijing, China; New Mexico State University, United States of America

## Abstract

Meta-analysis was performed for three major foliar diseases with the aim to find out the total number of QTL responsible for these diseases and depict some real QTL for molecular breeding and marker assisted selection (MAS) in maize. Furthermore, we confirmed our results with some major known disease resistance genes and most well-known gene family of nucleotide binding site (NBS) encoding genes. Our analysis revealed that disease resistance QTL were randomly distributed in maize genome, but were clustered at different regions of the chromosomes. Totally 389 QTL were observed for these three major diseases in diverse maize germplasm, out of which 63 QTL were controlling more than one disease revealing the presence of multiple disease resistance (MDR). 44 real-QTLs were observed based on 4 QTL as standard in a specific region of genome. We also confirmed the Ht1 and Ht2 genes within the region of real QTL and 14 NBS-encoding genes. On chromosome 8 two NBS genes in one QTL were observed and on chromosome 3, several cluster and maximum MDR QTL were observed indicating that the apparent clustering could be due to genes exhibiting pleiotropic effect. Significant relationship was observed between the number of disease QTL and total genes per chromosome based on the reference genome B73. Therefore, we concluded that disease resistance genes are abundant in maize genome and these results can unleash the phenomenon of MDR. Furthermore, these results could be very handy to focus on hot spot on different chromosome for fine mapping of disease resistance genes and MAS.

## Introduction

**Figure 1:**
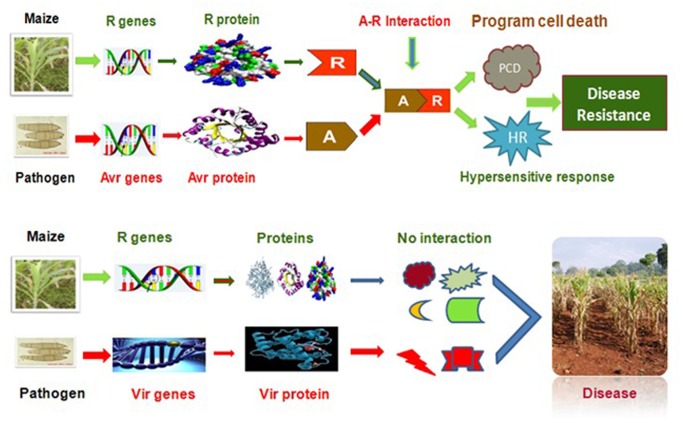
Interaction of the A-virulence and virulence protein with pathogen. The key-lock approach and the interaction of A-virulence and virulence proteins of the pathogen with the concern R-protein.

Plants and pathogens are continuously confronted with each other during evolution in a battle for growth and survival. In this rivalry plants have evolved a stunning array of structural, chemical, and gene-based defenses, designed to combat pathogens of different nature [1] and, so as the pathogens by developing new races. The overall destruction of maize diseases and the major diseases of maize crop has been documented [2]. The interaction of pathogens with the resistance genes are just like a key to lock approach, while the virulence genes in the pathogens can cause disease in its host regardless of the genetic architecture of the host plant (Figure 1). The basic concept underlying this phenomenon is the R-genes [3]. In maize and several other crops majority of R genes which had been investigated by gene cloning encode nucleotide binding site (NBS) and a leucine-rich-repeat (LRR) region. The NBS-LRR types of genes are abundant in all plant species [4] while it has been divulged that genome of maize include 109 NBS-encoding genes [5].

Usually, plant disease resistances can be classified into two broad categories: qualitative resistance conferred by a single resistance (R) gene and horizontal resistance or quantitative disease resistance (QDR), which are controlled by numerous genes of minor effect and are therefore called multigenic or genetically complex [6]. The qualitative resistance, mostly control by single major gene and is simply inherited but can be easily overcome by the racial evolution of concerned pathogens. On the other hand, resistance control by many genes, QDR provides more protection to the host plant as the pathogen strains will not be under selective pressure and loss of one or two genes from the bunch of genes will not leave the host completely susceptible [7]–[9]. The biggest problem in QDR is that the genes responsible for a specific disease resistance are mostly minor genes of small phenotypic effect. The total phenotypic variations control by these minor genes is usually very low and identifying these genes is a cumbersome and hard task. In short, QDR is conditioned by several genes and is race-non-specific, which is an effective tool against existing and novel races of pathogens. Broad-spectrum resistance and durable resistance to diseases are desirable for crop improvement. Meanwhile, finding all the genes for the most prevailing diseases in different crops at whole genome level is the primary issues of today’s agriculture. The study of quantitative disease resistance in maize is important because it is the most widely utilized form of resistance. However, very little is known about the physiological and molecular genetic basis of quantitative disease resistance. Dissection of QTL must be accomplished in order to lay the foundation for identification of the genes underlying the disease quantitative trait loci.

The other type of disease resistance is multiple diseases resistance (MDR) where one gene can control more than one disease [10]–[11] but this phenomenon has not been well explored. Zwonitzer et al [12] identified QTL for resistance to several diseases i.e. northern leaf blight (NLB), gray leaf spot (GLS) and southern leaf blight (SLB) in a maize recombinant inbred line (RIL) population, and evaluated the evidence for the presence genes or loci conferring MDR. These three diseases are potential threats to maize yield production and cause heavy losses all over the world. Highly significant correlations between the resistances to the three diseases were found [11].

Worldwide, the most damaging foliar diseases in maize are probably NLB, SLB and GLS. NLB developed and damaged seriously during 1971–5, causing about 50% yield loss SLB (*Helminthosporium maydis* race T) resulting in a loss of about 700 million bushels of corn in 1971. More recently the new *Helminthosporium* race was widely disseminated and was reported from most continents resulting in epiphytotics and severe losses in yield [2]. GLS is another most significant yield-limiting disease of maize worldwide, which was first found in Illinois, USA. In 1995, the reported yield losses due to GLS were as high as 50% in some U.S. maize fields [13]. The reported yield loss from this disease was 5–30% but in severe cases the losses may exceed 50%. Thus to solve the puzzle of MDR in maize, we elaborate the phenomenon with meta-analysis for these three important diseases at whole genome level in maize. More information in this field will open new era in the most complicated arena of resistance. Documentation of MDR genes would provide in-depth understanding into the evolution of pleiotropic effects and phenomenon that deliver quantitative resistance and allow for more strategic deployment of resistance genes in the development of unique cultivars. Already published article about NLB, SLB and GLS were used in this study to find out the hotspots in the whole genome of maize, and to figure out the QTL controlling all the three diseases. The purpose of this study is to summarize all the candidate genes related to these three diseases and build up the association between annotated NBS-LRR gene and mapped disease QTL, thus to find a reliable source of resistance.

## Materials and Methods

### Collecting and Reporting QTL Data for NLB, SLB and GLS

**Figure 2:**
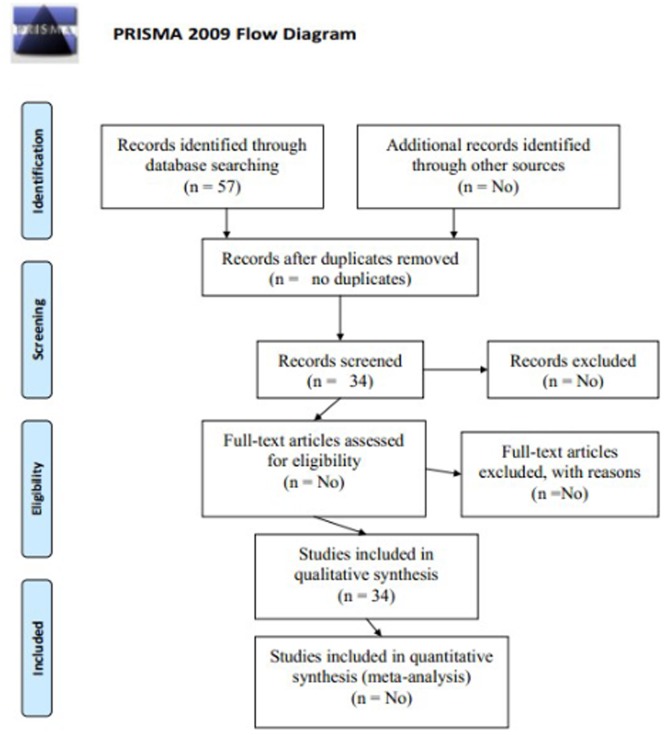
The PRISMA flow chart.

We collected the entire published article from Google scholar published about QTL mapping for the three foliar diseases of maize (NLB, SLB and GLS). The relative information of PRISMA check list (Table S4) and flow chart is given with all necessary information (Figure 2). The linkage map and QTL information for R^2^ of genetics effect, LOD value, linkage group and adjacent marker were required for the meta-analysis (Table 1). Furthermore, the software BioMercator V3.1 also needs the information about the QTL peak; its confidence interval (CI) and position for analysis. Numerous challenges were confronted in comparing the results from different published articles because all the studies were performed with many different populations and used different sets of molecular markers. Different scientist reported their results in different ways and some of the articles do not give the genetic map while in some LOD, CI or PVE were missing. With recent achievements in the field of molecular biology and statistical procedure it is possible to unite all the previous studies for meta-analysis and find several QTL for different diseases and even one QTL which can control all the three diseases. We used several formulas [14] to calculate different types of missing information. The missing CI for a specific QTL was calculated by the formula to infer the 95% CI for this QTL.

**Table 1:** The total number of QTL identified by different analytical methods in different population published literature for NLB, SLB and, GLS.

Genetic Material	Pop-size	Pop-type	Trait	QTL #	Method	Reference
DF20×LH146Ht	95	F2	NLB	1	LA	[36]
A619Ht2×W64A	124	F2	NLB	1	LA	[37]
B52×Mo17	150	F2∶3	NLB	10	SIM	[38]
W22Htn1×A619Ht2	95	BC1	NLB	2	LA	[39]
B52×Mo17	121	F2∶3	NLB	6	SIM	[40]
D32×D145	220	F3	NLB	13	CIM	[41]
Lo951×CML202	194	F2∶3	NLB	18	CIM	[42]
Lo951×CML202	194	F2∶3	NLB	16	CIM	[43]
Highland×lowland	196	F2∶3	NLB	2	CIM	[44]
IL731a×W6787	157	F2∶4	NLB	14	LA	[45]
Z3×P138	230	F2∶3	NLB	8	SIM	[46]
Ki14 × B73	109	RIL	NLB	7	CIM	[47]
B73 × Mo17	302	RIL	NLB	25	CIM	[48]
NAM	5000	RIL	NLB	29	CIM	[49]
ADENT×B73rhm	139	F2∶3	SLB	5	ANOVA	[7]
Highland×Lowland	196	RIL	SLB	1	CIM	[44]
B73×Mo17	158	RIL	SLB	11	CIM	[50]
B73×Mo17	192	F3∶4	SLB	10	CIM	[51]
B104×NC300	133	RI	SLB	13	CIM	[52]
B73×Mo17	298	RIL	SLB	23	CIM	[53]
B73×B52	186	RIL	SLB	7	CIM	[18]
H99×B73	142	RIL	SLB	2	CIM	
Ki14×B73	109	RIL	SLB	9	CIM	[12]
B73×CML254	120	RIL	SLB	6	CIM	[54]
B97×Ki14	214	RIL	SLB	4	CIM	
CML254×B97	126	RIL	SLB	9	CIM	
NAM	5000	RIL	SLB	30	RA	[30]
T14×T4	330	F2∶3	SLB	18	CIM	[55]
ADENT × B73rhm	139	F2∶3	GLS	33	SIM	[56]
FR1141 × O61	301	BC1S1	GLS	16	CIM	[57]
Proprietary F2	230	F2	GLS	7	CIM	[58]
Va14 × B73	239	F2∶3	GLS	12	SIM	[59]
VO613Y × Pa405	100	F2∶4	GLS	2	CIM	[19]
B73×Mo17	288	RIL	GLS	6	CIM	[18]
Ki14×B73	117	RIL	GLS	8	CIM	[12]
L30×L31	240	F2	GLS	5	SIM/RA	[60]

Note: Pop-size (Total number of individuals in the population), Pop-type (Type of population used), QTL # (Number of QTL/s identified in the concern study), NLB (Northern Leaf Blight), SLB (Southern Leaf Blight) and GLS (Gray Leaf Spot), LA (Linkage Analysis), SIM (Simple Interval Mapping), CIM (Composite Interval Mapping), ANOVA (Analysis of variance), RA (Regression Analysis).

(1). CI = 530/(N×R^2^)

(2). CI = 163/(N×R^2^)

CI is the confidence interval, N is the number of population, R^2^ is the genetics effect. The (1) formula was suitable for backcross (BC) and F2 population, and the (2) best suited for recombination inbred lines (RIL) populations.

If only the R2 information was given in the article then we calculated the LOD by the following formula.

LOD = R^2^/1.5.

All the necessary information about the QTL, required for the meta-analysis was calculated and the data were subjected to analysis.

### QTL Projection and Meta-analysis for NLB, SLB and GLS QTL

QTL projection and Meta-analysis were performed by the software BioMercator V3.1 of each chromosome [15]–[16]. The IBM 2008 was used as the reference map, downloaded from www.maizeGDB.org/. The QTL projection was performed with projection command to the reference map in the manual. Prior to the QTL projection, we find the same marker in literature about NLB, SLB and GLS and in the reference map. If the marker information was not synchronized, i.e. have no similarity between the markers, we used BLAST the primer information of the QTL to B73 AGP2 genome (www.maizesequence.org/) for finding the adjacent flanking markers in the reference map. The remaining QTL having no consensus marker and no information about the physical position of markers were excluded from the analysis. Consequently, the arranged QTL information and reference map were utilized for the QTL projection with the command projection in BioMercator 3.1 to get the reference map QTL information.

Meta-analysis was used to figure out the total number of “real QTL” in our analysis for the three diseases. The “real QTL” rely on the QTL information identified based on Akaike-type criteria values (AIC) value. The relative regions with less than three QTL were not considered for meta-analysis. We get the 10 times simulation of AIC value, while the minimum AIC value was according to the “real QTL” by the command “QTL *Meta analyses*”. These results give us new information about the “real QTL” its peak position and CI.

### NBS-LRR Gene Family Prediction

The total number of NBS-LRR genes identified in maize genome was projected in this study. A full set of candidate disease resistance genes encoding NBSs (referred to as NBS-encoding genes) was used from the complete maize genome [5]. Cheng et al [5] have observed that a total of 109 NBS disease resistance genes harbors within the 2300-Mb maize genome and of these, 107 were regular and two were non-regular NBS-encoding genes. We find out physical position of flanking sequence of all the 107 NBS genes based on the B73 reference genome (www.maizeGDP.org). After the meta- analysis, we compare the physical position of these NBS encoding genes with the identified QTL. The purpose is to find out that how many NBS encoding genes can be identified in our analysis. Furthermore, the most important genes for the *Ht1* and *Ht2* genes were also predicted to confirm these genes and locate to any real QTL for future use in molecular breeding.

## Results

### Disease QTL Collection

The QTL information was collected from the web and the IBM 2008 map was used as a reference. Different types of bi-parental segregating populations derived from primary or advance self-pollination or backcross of maize germplasm with diverse geographical origins were used in maize disease QTL studies (Table 1). Several diverse genetic background germplasm and different population size for maize disease QTL mapping ranges from 95 to 5000 were used. Different analytical approaches have been used for different studies and several scientists have observed more than 30 QTL in a single study for specific disease (Table 1). A total of 389 QTL were observed for these three major diseases in maize and only about 36 studies were reported (if we take each row in table 1 as a single study). Different genetic backgrounds have been used in all the studies but one thing is clear that most of the scientists used B73 as parent in their research. We used the B73 as a reference map and it can be more appropriate to find the exact position of maximum QTL and true genes controlling disease resistance in maize.

### Distribution of QTL on Different Chromosome

**Figure 3:**
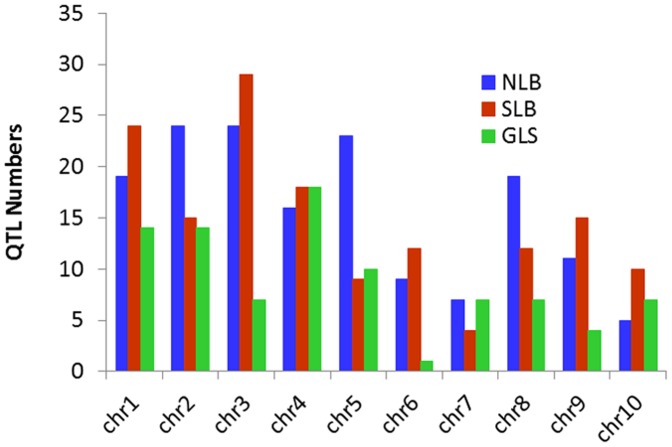
Distribution of QTL for three diseases on all the 10 chromosomes of maize. This graph shows the total number of QTL for NLB, SLB and GLS on the entire chromosomes and each chromosome harbored resistance genes at a specific region. NLB (Northern Leaf Blight), SLB (Southern Leaf Blight) and GLS (Gray Leaf Spot).

The total numbers of QTL for all these three diseases were randomly distributed on all 10 chromosomes (Figure 3) but each chromosome harbored resistance genes at a specific region. Maximum number of QTL for NLB was observed on chromosome 3 while the chromosome 10 possessed the least number of resistant QTL. More than 20 QTL were observed on chromosome 2, 3, and 4 regarding NLB. The chromosome 8 was also important for NLB but the QTL for the rest of two diseases were less on this chromosome than chromosome 4. Maximum QTL for SLB was observed on chromosome 3 and chromosome 1 was also important harboring more than 20 QTL. The entire nine chromosomes possessed more than 5 QTL each for SLB except chromosome 7, which were less among all. Among the three diseases the minimum number of QTL were observed for GLS and maximum QTL were observed on chromosome 4. Chromosome 6 was not revealed to be very important for GLS as minimum numbers of QTL for this disease were observed on this chromosome. Chromosome 4 was more consistent for all the three diseases and more than 15 QTL was observed for each disease. However, totally 12 NBS-encoding genes were observed on this chromosome but still not determined in any study. Maximum QTL for NLB and SLB were observed on chromosome 3 but the GLS QTL was less than 10. The numbers of QTL on chromosome 10 were less than 10 for each of the disease but the numbers of NBS-LRR genes were the maximum on this chromosome (Table S1). Totally 28 NBS-LRR genes were observed on chromosome 10 and 12 genes on chromosome 3. The numbers of QTL for the two diseases (NLB and SLB) were the highest on this chromosome. Totally 7 NBS genes were observed on chromosome 8 while chromosome 9 harbored only one CC-NBS-LRR gene and chromosome one have 9 NBS genes.

### Clustering and Multiple Disease Resistance QTL

**Figure 4:**
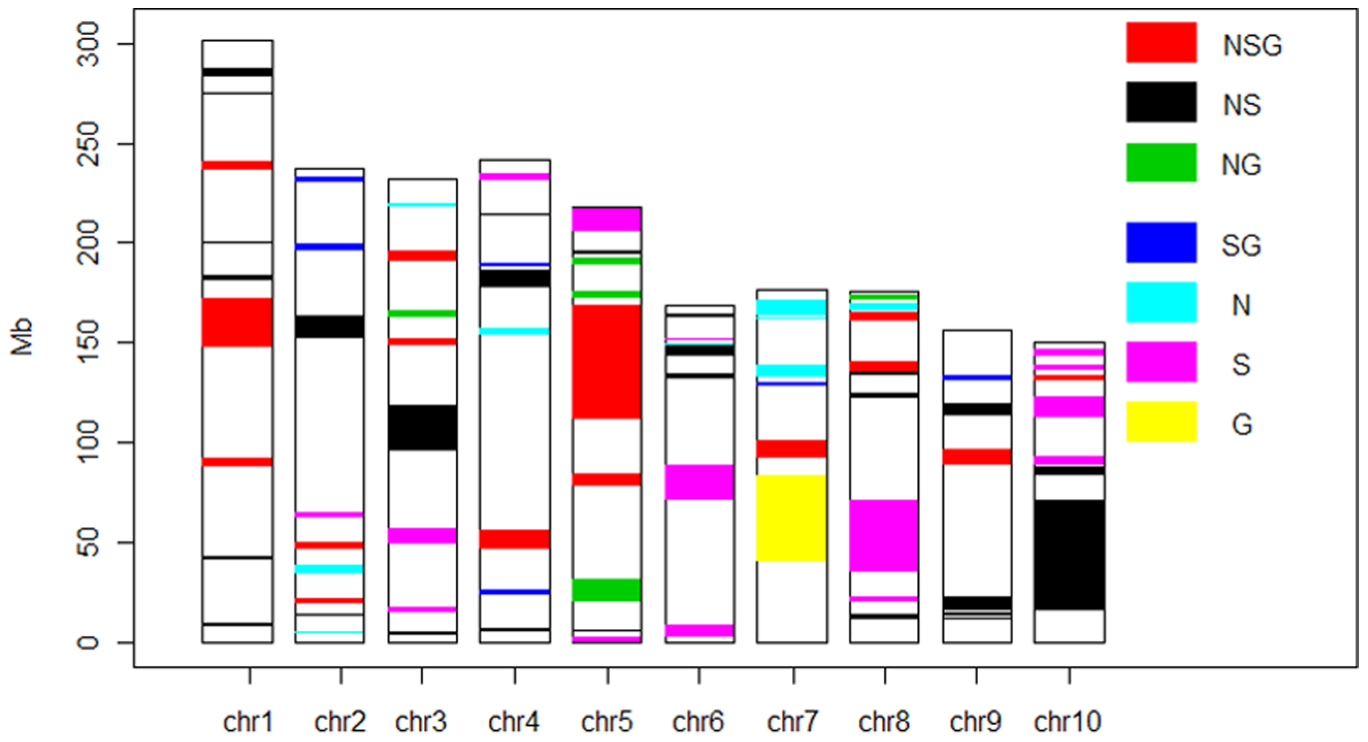
Heat map showing the intensity and Clustering of different types of QTL in maize. The Heat map showed the clustering of QTL for NLB, SLB and GLS in maize genome. Different diseases were depicted with different colors.

**Figure 5:**
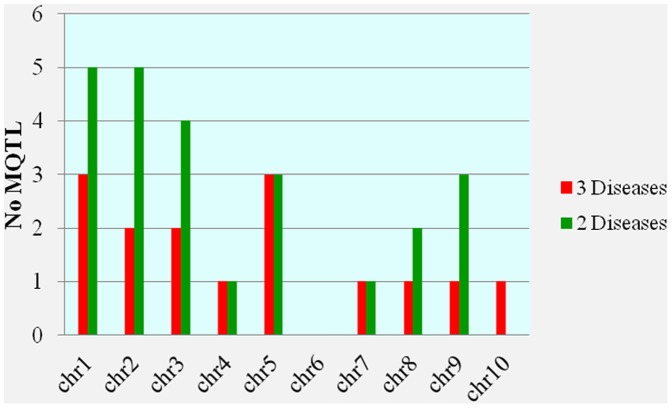
Clustering of QTL on different chromosomes for more than one disease. Number of multiple disease resistance QTL on different chromosomes.

Usually the disease resistance QTL clustered on different chromosome as observed by many scientists [10]–[11]. We observed several QTL in the same location on different chromosome (Figure S1, S2, S3, S4, S5) and mostly they are very close to each other. The distribution of disease resistance cluster is random in maize genome and chromosome 1 and 3 have a lot of QTL in very narrow regions. Multiple disease resistance is of prime importance to redeem the losses by concerned pathogens and we find several QTL, which can easily be used in marker assisted selection to control many diseases at the same time. The hot spot for MDR is chromosome 5, where a big cluster was observed in the heat map showing different types of QTL in different colors (Figure 4; Table S2). The biggest cluster for NSG, NS, S, and G were observed on chromosomes 5, 10, 8, and 7, respectively (Figure 4). The clustering of disease QTL on different chromosome in specific regions could be the main source of the most unmitigated source of multiple disease resistance. Therefore, we examined the total genome of maize to find out the total number of multiple disease resistance QTL (MQTL) for different disease (Figure 5). Chromosome 1, 2 and 3 were very important for different diseases and harbored maximum number of QTL. Five MQTL was observed both on chromosome 1 and 2 while chromosome 3 has four MQTL. On chromosome 8, we observed only one MQTL for three diseases while 2 MQTL were for two diseases. Chromosome 4, 7, 8, 9, and 10 harbored one MQTL for all the three disease while the number of meta-QTL controlling more than one disease was different. On chromosome 5 the number of MQTL for two and three diseases was the same and the number of 3 disease QTL was the same with chromosome 1 (Table S2).

### Meta-analysis and Real QTL

**Figure 6:**
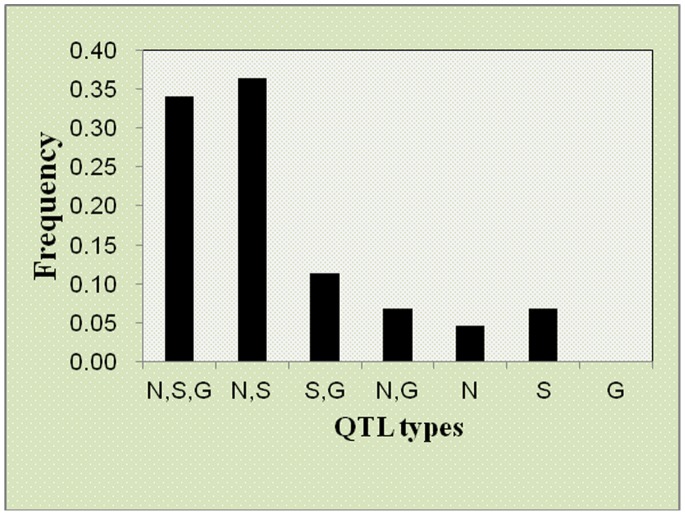
Frequency distribution of 5 different types of QTL in maize genome. Almost 35% real QTL were observed for MDR QTL which control all the three diseases and above 35% real QTL control the two diseases, which are recently under extensive research i.e. NLB and SLB.

The meta-analysis revealed lump sum of 88 QTL for the three diseases distributed on different chromosomes. Totally 10 meta-QTL were observed on 5 chromosomes (2, 3, 4, 5 and 9) while 9, 8, 7 and 6 QTL were located on chromosomes 1, 6, 10 and 7, respectively. We analyzed the different meta-QTL types for all the three diseases in different combination and observed maximum MQTL for NLB plus SLB. The phenomenon of these two diseases may be more similar and our results showed that maximum QTL were controlling these diseases. Figure 6 showed the different types of QTL and their respective frequency for different diseases. Regarding the real QTL the number was randomly distributed in different chromosomes and totally 44 real QTL were observed in the maize genome. The first three chromosomes harbored 8 real QTL each, while the minimum real QTL were observed on chromosome 6 and 10 (Table S2). We inferred the CI of each QTL in our analysis and observed a significant low interval for several real QTL, among which the lowest was 0.9 cM on chromosome 3 and this real QTL was for SLB (Table S3). Chromosome 8 possessed cluster of QTL and also have a significant real QTL for all the three diseases with a CI less than 5. The maximum numbers of NBS-encoding genes were present on chromosome 10 and we observed a real QTL of all three diseases with a low CI. The real QTL was also randomly distributed on different chromosomes and maximum number was observed on chromosome 1, 2, 3, and 5. Almost 35% real QTL were observed for MDR QTL which control all the three diseases and above 35% real QTL control the two diseases, which are recently under extensive research i.e. NLB and SLB (Figure 6).

### Associations between QTL and NBS-LRR Genes

**Figure 7:**
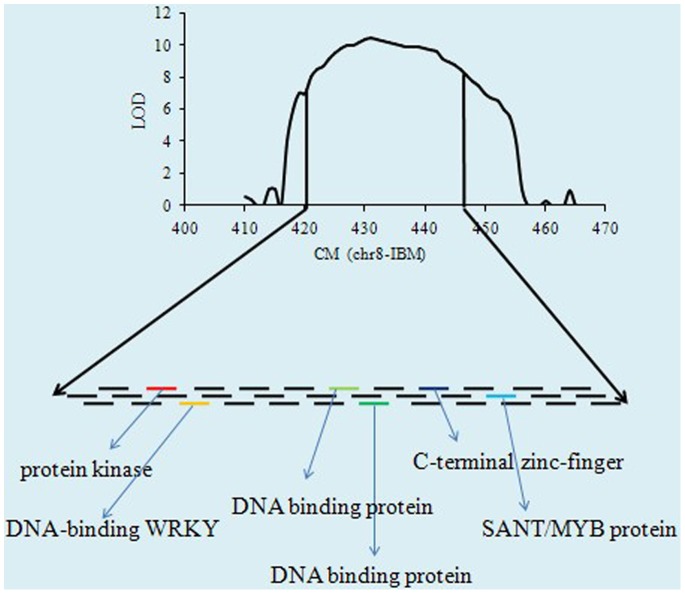
The real QTL on chromosome eight and some other genes annotated in this region. Among these NBS gene family two genes was observed in the same QTL on chromosome 8 and 10. The *Ht2* gene was also observed on chromosome 8 along with these two NBS genes.

**Figure 8:**
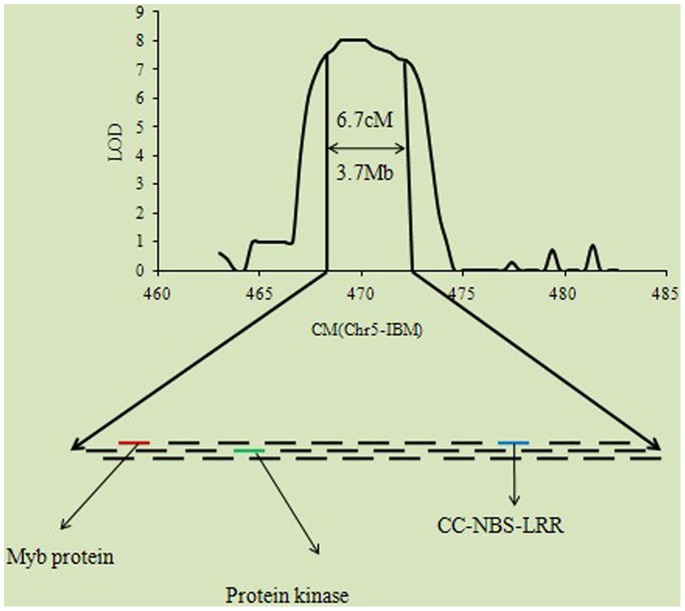
QTL and gene information on chromosome five. The information on chromosome 5 is also provided and some disease resistance genes were observed along with a NBS gene.

**Figure 9:**
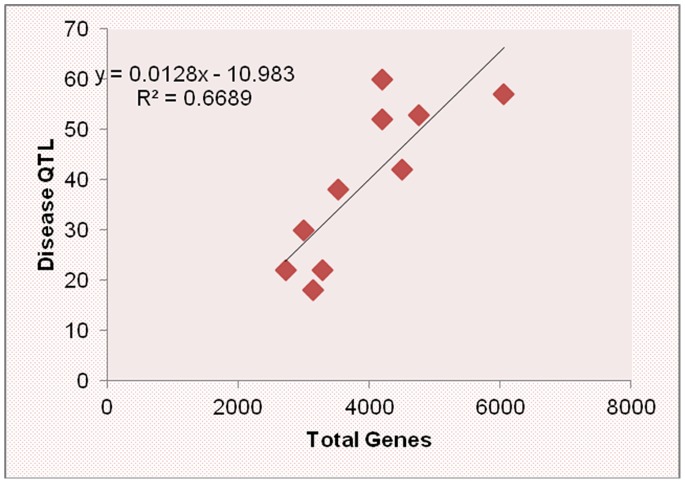
Relationship between the number of disease QTL and total genes per chromosome of maize. The total QTL for diseases have a significant relationship with the total number of genes per chromosome in maize genome based on the reference genome B73. The total gene number is from the reference genome of B73.

The NBS- encoding genes observed in several real QTL showed that we can focus on these regions in future for identifying resistance sources which are more powerful to stop destruction of maize by pathogens. We observed chromosome 1, 2, 5, 7, 8, 9, and 10 harbored the NBS-encoding genes which control these diseases (Table S3). We observed 14 genes out of 107 NBS-encoding genes, which indicate that several other gene families may be involved in protecting maize against these three diseases. The other point which is obvious from this analysis is that the maize NBS-encoding genes were not observed for these diseases and more work is required for exploring the detail role of NBS genes in disease resistance. Chromosome 1, 5, 7 and 8 harbored two NBS-LRR genes each while chromosome 2, 3, and 9 possesses one NBS gene each. The rest of three genes were observed on chromosome 10. Among these NBS gene family two genes was observed in the same QTL on chromosome 8 and 10. The *Ht2* gene was also observed on chromosome 8 along with these two NBS genes (Figure 7). So this gene may be the possible candidate gene and can be one of these NBS genes. Several other genes were also observed in this region like protein kinase, DNA-binding WRKY and SANT/MYB protein in this region showing that this chromosome is extremely important for these three diseases (Figure 7). The information on chromosome 5 is also provided and some disease resistance genes were observed along with a NBS gene (Figure 8). Furthermore, the total QTL for diseases have a significant relationship with the total number of genes per chromosome in maize genome based on the reference genome B73 (Figure 9). The total gene number is from the reference genome of B73 http://www.maizegdb.org/gene_model.php#gm6.

## Discussion

The history of maize diseases has revealed that diseases have the potential to cause significant losses to the total production [17]–[18]. A thorough understanding of disease resistance will open the gateway of improving the genetic basis of maize and high resistance germplasm can be produced for increasing the total production of world’s leading cereal crop.

The most proper way to identify QTL for MAS is to compare the known QTL in literature for their consistent location and effect across diverse environment in different genetic backgrounds. The QTL identified in the same location in different articles across diverse environment explaining high phenotypic variation can be used meritoriously for MAS. QTL meta-analysis is refinement of QTL positions on the consensus map [19] and an approach to detect consensus QTL across unlike studies, to confirm QTL effects different across environments and multiple genetic backgrounds. This method provides decision rules based on an adjusted Akaike Information Content (AIC) criterion to select the QTL model on each chromosome and determine the number of real QTL that best fits the results on a given linkage group. The meta-analysis groups the QTL detected in independent experiments that correspond to the same QTL and finally provides a consensus estimation of QTL positions with the smallest confidence interval (CI) and large effect on the specific trait. We identified several QTL controlling all the three diseases. Many of these QTL have a very low CI and they can be used for revealing the genetic architecture of maize regarding these diseases. We found out several important genes like *Ht1* and *Ht2* underlying in these QTL for validation of the results. The information obtained from this study can be used for future analysis and help to identify candidate loci/genes for different diseases in maize underlying these regions. These real-QTLs could be the hotspot of several diseases and target oriented studies can be performed for exact identification of resistance genes.

### Evidence of Multiple Disease Resistance

Disease resistance genes are the only environmentally friendly source of controlling several diseases in maize. We have observed several disease resistant QTL in maize genome controlling more than one disease. The total number of these QTL in our analysis was 63 for all the three diseases in different combination (NSG, NS, NG, and SG). The number of MDR QTL was the highest among the total number for NS (34), while for NSG 17 MDR were observed indicating the presence of multiple disease control in maize. These analyses implied that in maize genome the disease are controlled by same QTL in several cases and these QTL might be evolved at the same time during the process of selection. Wisser et al [20] observed that plants are attacked by several pathogens of diverse taxonomic groups, such that genes providing MDR are expected to be under positive selection pressure. The germplasm used in different studies showed astonishing diversity as each study was performed in different places with different kind of germplasm and environments. The sources of resistance may be combined from different germplasm to explore the phenomenon of disease resistance in detail and show the evolution of disease resistance genes. This idea can also be supported by the presence of two NBS-encoding genes in the same QTL on chromosome 1, 8, and 10. As we have observed only a few NBS encoding genes for these three diseases but still, they are in one QTL and very near to each other. Evidence that MDR genes exist in plants includes the detection of clusters of quantitative trait loci for different diseases [10], [21] and the identification of induced gene mutations that affect plant responses to infection with different pathogens [20], [22]. The field of disease resistance is very wide and still the total numbers of disease resistance genes are not explored. To find out the total number of genes in maize genome, a large population with ultra-high density marker will be required. McMullen and Simcox [23] observed that the disease resistance genes were clustered in maize and several important bin positions had been mentioned by other scientists [11], [21]. On chromosome 3, we observed several cluster and maximum MDR QTL indicating that the apparent clustering could be due to genes exhibiting pleiotropy for MDR. Wisser et al [20] used a multivariate test statistic and found a glutathione S-transferase (GST) gene, which was significantly associated with modest levels of resistance to NLB, SLB and GLS. Michelmore and Meyers [20] also observed that most of the resistance genes are in cluster. These clusters might be the hotspot of disease resistance genes. The phenomenon of disease resistance is not well explained at gene level in plant but *Ghd7* [24] has been observed to play a major effects on an array of traits in rice, including number of grains per panicle, plant height and heading date. In human being and several other organisms the pleotropic effect is perfectly explained at gene level [25]. For instance, Marfan syndrome is a disorder in humans in which one gene is responsible for a constellation of symptoms, including thinness, joint hypermobility, limb elongation, lens dislocation, and increased susceptibility to heart disease. Similarly, mutations in the gene that codes for transcription factor TBX5 cause the cardiac and limb defects of Holt-Oram syndrome, while mutation of the gene that codes for DNA damage repair protein NBS1 leads to microcephaly, immunodeficiency, and cancer predisposition in Nijmegen breakage syndrome [25]. Another example of pleiotropy in humans is phenylketonuria (PKU), where defect in the single gene that codes for this enzyme results in the multiple phenotypes, including mental retardation, eczema, and pigment defects that make affected individuals lighter skinned [26]. Our results showed that most of the time diseases resistance genes were in the same region on different chromosomes and they might be under strong selection pressure in the maize breeding throughout the history. The resistance genes might be evolved from the same source but extensive work will be required to find the genetic evidence of one gene controlling more diseases at the same time or differentially performed at different time during the life span of maize. The phenomena of these three diseases (NLB, SLB, and GLS) might followed the same genetic pathway and large data will be required to explore that why certain QTL control more than one disease at a time or they can perform at different stages to control another disease.

### Molecular Breeding for Disease Resistance and the Role of Meta-analysis

Since the early days of the 20th century, classical breeding for manipulating different traits in plant has been a primary method for improvement and enhancing the ability of crop against different stresses. Revealing the genetic architecture of different crops different approaches have been used by different scientist [27] throughout the world and meta-analysis will increase the chances of gene identification and recognition of hotspot for MDR and distinct disease resistance QTL. The scientific community has been trying to figure out an easy, convenient and economic approach for finding genes controlling the important traits in crop plants.

In traditional QTL mapping, about 20 cM is the limited confidence interval from the whole genome [28]. Now, with the fast growing achievements in the field of molecular biology and genetics, involvement of high-density markers and bin-map make the process of mapping easy and more accurate [29]. Furthermore, the meta-analysis revealed several QTL with very low CI and it can be suggested that several QTL regions can be reduced significantly through meta-analysis. It is obvious from our analysis that maize possessed several MDR QTL and positional cloning can be the best approach taking some well-known genes based on annotation and other properties [11]. The MDR QTL can be examined thoroughly, while the reference genome and annotation can be used for collecting the preliminary information about genes cloning. This phenomenon can significantly reduce time and resources of molecular breeding in maize against many diseases.

Keeping in view the global diversity of maize, the reference genome availability can be handier but only the B73 genome is not enough as plenty of genes cannot find physical position and identification of exact genes for disease resistance is difficult [2]. There should be a wide range of references, so that all the information can be easily obtained. Therefore, a disease resistant line as a reference genome is extremely important. Most of the articles published about different diseases give less information about all the identified QTL and most of the genes are not characterized. As mentioned earlier, maize is affected by more than 100 pathogens [2], so an entire genetic pool must be available which represents the entire genetic variability or the available diversity of maize. The wild maize can be a good gene bank for the breeding community because of the high genetic diversity in maize crop. Providing more information about different genetic phenomenon will open the door of novel fields in molecular breeding for disease resistance and in the near future the maize crop will be manipulated in desirable direction with fingertips.

All the above techniques can be used together to identify the QTL for all the diseases in maize and a combine source of resistance can be found out to stop the reduction in yield. The meta-analysis is a nice approach to collect all the QTL from the genome and identify a hotspot for different diseases. This information can be significantly used in increasing the pace of molecular breeding. The meta-QTL was abundant in maize and we observed more than 60 MDR for NLB, SLB, and GLS. The CI of different QTL was very low and certain QTL can be focused to identify the disease resistance genes. Our analysis revealed that meta-analysis will play a key role in the near future for identification of disease resistance genes and improve the process of QTL mapping providing a firm foundation for molecular breeding.

### Implication of Meta-analysis in Marker Assisted Selection (MAS)

Classical genetic and molecular data showed that genes determining disease resistance in plants are frequently clustered in the genome [27]. Recently a great progress has been observed via high throughput technologies, which in turn have enabled ground-breaking discoveries in plant sciences. Once the markers associated with economical traits have been identified then the MAS can be used to manipulate maize for different diseases. The development of highly diverse population and high density markers are extremely handy to identify genes for economically important traits. The high density markers are now in use to construct ultra-high density bin map with global collection of maize to divide the genome into possible small fragments. The flanking markers of each bin can be used after meta-analysis to identify genes in the concerned QTL. We have observed more than 80 disease resistance QTL and provided basic information about the genetic basis of disease resistance in maize. Disease resistance is a complex phenomenon and several diseases cannot be studied in one experiment. The inoculation of one population by several pathogens is not possible and a single locus for different diseases is hard to identify in a single study. The easiest approach is to combine the data from different studies about different disease and to find if the same region for different diseases can be observed or not. Once the QTL controlling more than one disease is identified then the available marker information can be used for MAS. Instead of developing a hybrid having resistance to one disease the meta-analysis approach could be handy for developing MDR varieties/hybrids. The QTL on chromosome 5 and 8 harbored some important genes for these diseases and also annotated genes previously reported to play role in disease resistance [8], [30]. While annotating different genes for resistance, they also observed the association of proteins kinase for resistance to NLB and SLB, respectively. A number of genes encoding WRKY proteins have been isolated from different plants, with certain that are induced rapidly by pathogen infection, or treatment with pathogen elicitors [31]–[32]. Investigating the role of WRKY DNA binding protein in the regulation of resistance gene expression, Yu et al [33] provided solid evidence that certain WRKY genes play an important role in regulating of the plant defense response and induced disease resistance by regulating defense proteins with direct or indirect antimicrobial activities. Most the genes in these identified QTL are hypothetical protein and need to be studied for their possible role in disease resistance. The ultimate goal of our analysis was to provide breeder ready marker for MAS to upgrade the economic value of maize and assure the global food safety.

### The NBS-LRR Genes in Disease Resistance

Nucleotide-binding site (NBS) disease resistance genes play an integral role in defending plants from a range of pathogens [5]. 109 NBS-encoding genes were identified based on the complete genome sequence of maize (*Zea mays* cv. B73). These genes were classified into four different subgroups, and then characterized according to chromosomal locations, gene duplications, structural diversity and conserved protein motifs [5]. We used these genes in our analysis to find the location of these NBS-encoding genes in the meta-analysis possibly in the real-QTL regions for these diseases. Totally, 14 NBS-encoding genes were identified and located on different chromosomes, distributed randomly on several chromosomes. Two genes from two groups were identified on chromosome 1 in the same QTL which control all the three diseases and having a low CI value (12.8 cM). This region was even smaller than the traditional QTL mapping (20 cM) and we can easily come close to the primary genes of disease resistance. These genes reside between the flanking markers (TIDP5752- AY112092) and the physical positions were from 454.7 to 467.5. The peak positions of these genes were also given and this basic information might be targeted for increasing the level of resistance in maize against these diseases. Similarly the real-QTL on chromosome 8 between the flanking markers (gpm685-umc1933) also controlled the three diseases, and was considered as a MDR QTL harboring two NBS-encoding genes of the same group. Chromosome 8 has been widely mentioned by several scientists to be involved in disease resistance and harbored a lot of resistance genes for all the diseases [11]. 11 out of these 14 NBS-encoding genes were CC-NBS-LRR encodes for an N-terminal coiled-coil (CC) domain- nucleotide binding site-leucine-rich repeats. These genes were consistently observed to have an integral part in protein–protein interactions and signaling [5], [34]–[35]. The possible reason for this phenomenon may be that these CC-NBS-LRR genes are providing signals to the basic resistance genes for activation at the time of invasion or infection of pathogen to stop yield losses. The *Ht2* gene was also identified on chromosome 8 in a real-QTL controlling NLB. Chung et al [11] also observed the *Ht2* genes on chromosome 8 bin 8.06 controlling NLB and was observed to be major gene for disease in maize. Our results suggested that the resistance genes were clustered and also specific QTL could control diseases providing proper evidence of MDR. More than one NBS-encoding gene was also present in one QTL and several genes were controlling more than one disease, even there is only one NBS-encoding gene identified in the specific QTL. Revealing the total number of NBS-encoding genes could increase the chances for super resistance maize germplasm as Jones and Dangl [3] showed that the most of the R genes which had been investigated by gene cloning encode nucleotide binding site (NBS) and a leucine-rich-repeat (LRR) region. Furthermore, we concluded that the NBS genes are not the only gene family responsible for disease resistance and several other genes family might play a key role in controlling diseases. The phenomenon of disease is not well elaborated and extensive work is required to explore the total number of resistance genes and gene families and categorize them into different groups based on annotation and their function.

## Supporting Information

Figure S1 Disease resistance QTL on chromosome 1 & 2. The Chromosomal distribution of disease resistance QTL on chromosome 1 and 2 with their possible flanking markers are given and we observed that distribution of disease resistance cluster is random in maize genome. Several QTL in the same location on different chromosome and mostly they are very close to each other.
(TIF)

Figure S2 Disease resistance QTL on chromosome 3 & 4. Chromosomal distribution of disease resistance QTL on chromosome 3 and 4 with their possible flanking markers.
(TIF)

Figure S3 Disease resistance QTL on chromosome 5 & 6. Chromosomal distribution of disease resistance QTL on chromosome 5 and 6 with their possible flanking markers.
(TIF)

Figure S4 Disease resistance QTL on chromosome 7 & 8. Chromosomal distribution of disease resistance QTL on chromosome 7 and 8 with their possible flanking markers.
(TIF)

Figure S5 Disease resistance QTL on chromosome 9 & 10. Chromosomal distribution of disease resistance QTL on chromosome 9 and 10 with their possible flanking markers.
(TIF)

Table S1 The total number of NBS-LRR genes family along with their description in maize genome and the physical position according to IBM2-2008.
(DOC)

Table S2 Description of all the QTL observed during the meta-analysis and the number of real QTL.
(DOC)

Table S3 Identified NBS-encoding genes, *Ht1*, and *Ht2* genes in the meta-analysis along with the physical position on different chromosomes responsible for disease resistance.
(DOC)

Table S4 The relative information of PRISMA check list. The study is a meta-analysis for several diseases and all the information is given on the respective mentioned page.
(DOC)
