# Supplementary figures and images for: Evidence of Multiple Disease Resistance (MDR) and Implication of Meta-Analysis in Marker Assisted Selection
Source: PLoS One. 2013 Jul 10;8(7):e68150. doi: 10.1371/journal.pone.0068150 (PMC3707948; doi:10.1371/journal.pone.0068150)

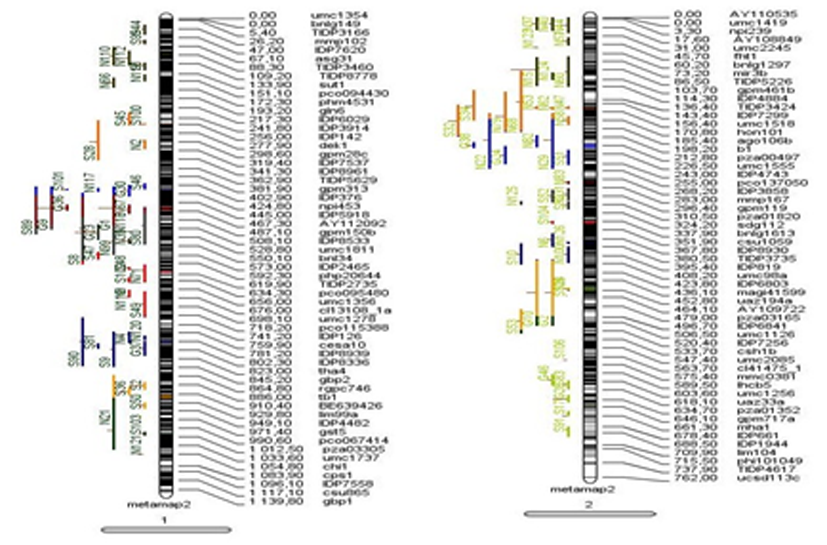

Supplement: Figure S1 — Disease resistance QTL on chromosome 1 & 2. The Chromosomal distribution of disease resistance QTL on chromosome 1 and 2 with their possible flanking markers are given and we observed that distribution of disease resistance cluster is random in maize genome. Several QTL in the same location on different chromosome and mostly they are very close to each other. (TIF) [file pone.0068150.s001.tif]

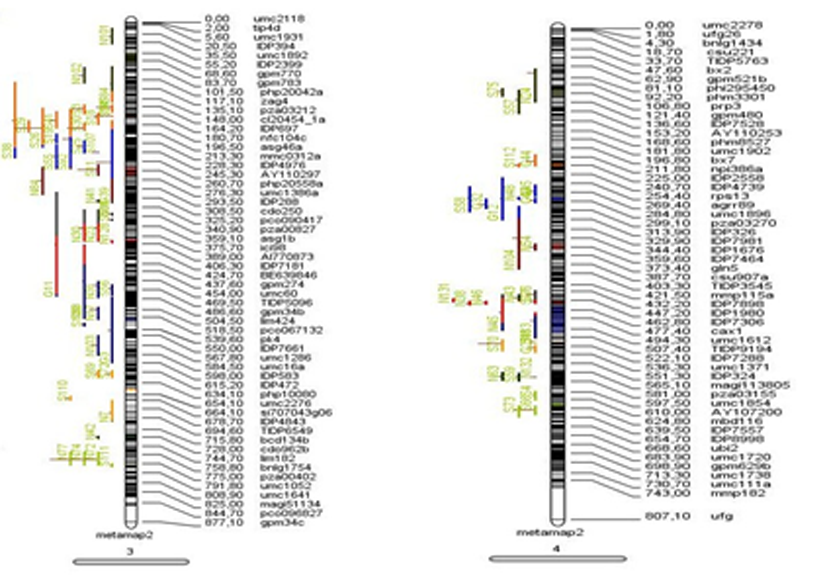

Supplement: Figure S2 — Disease resistance QTL on chromosome 3 & 4. Chromosomal distribution of disease resistance QTL on chromosome 3 and 4 with their possible flanking markers. (TIF) [file pone.0068150.s002.tif]

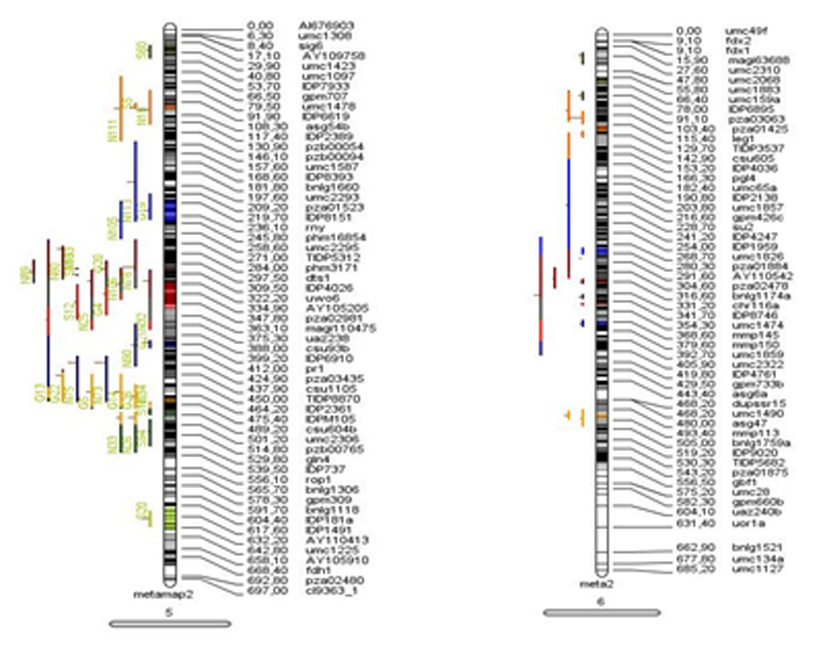

Supplement: Figure S3 — Disease resistance QTL on chromosome 5 & 6. Chromosomal distribution of disease resistance QTL on chromosome 5 and 6 with their possible flanking markers. (TIF) [file pone.0068150.s003.tif]

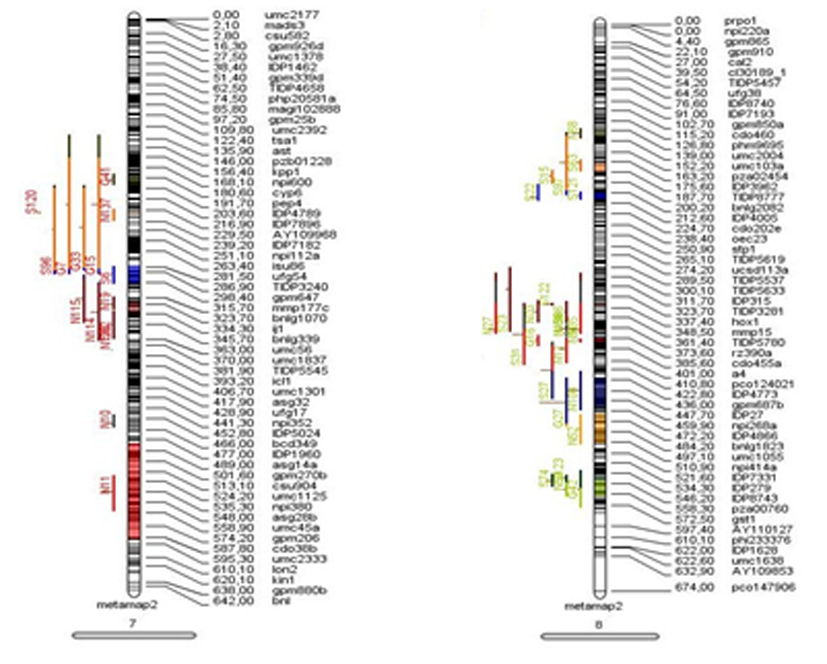

Supplement: Figure S4 — Disease resistance QTL on chromosome 7 & 8. Chromosomal distribution of disease resistance QTL on chromosome 7 and 8 with their possible flanking markers. (TIF) [file pone.0068150.s004.tif]

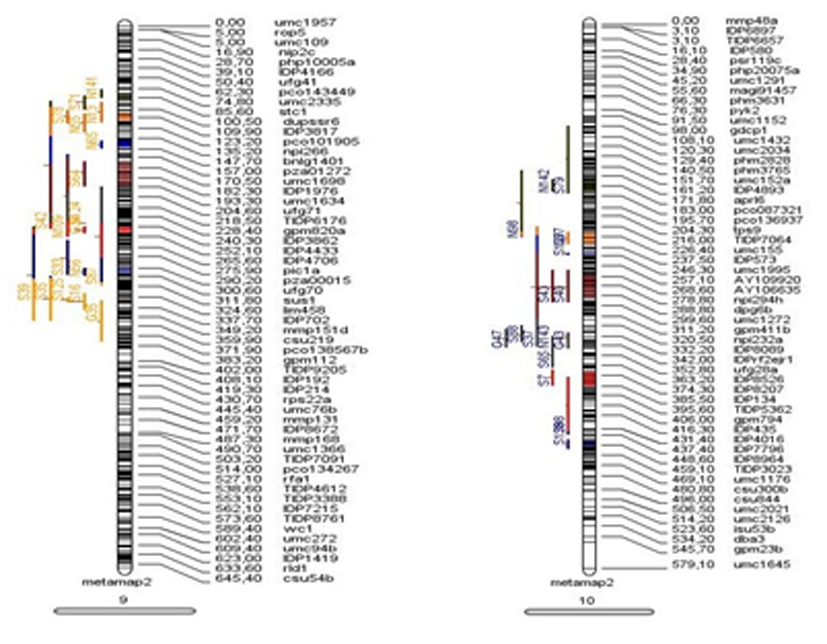

Supplement: Figure S5 — Disease resistance QTL on chromosome 9 & 10. Chromosomal distribution of disease resistance QTL on chromosome 9 and 10 with their possible flanking markers. (TIF) [file pone.0068150.s005.tif]
