# Supplementary material for: Evidence of Multiple Disease Resistance (MDR) and Implication of Meta-Analysis in Marker Assisted Selection
Source: PLoS One. 2013 Jul 10;8(7):e68150. doi: 10.1371/journal.pone.0068150 (PMC3707948; doi:10.1371/journal.pone.0068150)
Supplement: Table S1 — The total number of NBS-LRR genes family along with their description in maize genome and the physical position according to IBM2-2008. (DOC) [file pone.0068150.s006.doc]

Table S1 The total number of NBS-LRR genes family along with their description in maize genome and the physical position according to IBM2-2008

| Gene | Gene Name | Gene ID | Gene type | Chr | L. Pos | R. Pos |
| --- | --- | --- | --- | --- | --- | --- |
| 1 | ZmNBS67 | GRMZM2G136513_P01 | CC-NBS-LRR | 1 | 35,132,532 | 35,136,898 |
| 2 | ZmNBS61 | GRMZM2G033533_P01 | CC-NBS-LRR | 1 | 52,624,135 | 52,628,655 |
| 3 | ZmNBS71 | GRMZM2G443525_P01 | CC-NBS-LRR | 1 | 164,086,816 | 164,092,155 |
| 4 | ZmNBS107 | GRMZM2G443525_P02 | NBS-LRR | 1 | 164,089,066 | 164,092,155 |
| 5 | ZmNBS58 | GRMZM2G461269_P01 | CC-NBS-LRR | 1 | 180,332,681 | 180,338,561 |
| 6 | ZmNBS102 | GRMZM2G322748_P01 | NBS-LRR | 1 | 196,691,877 | 196,695,603 |
| 7 | ZmNBS31 | GRMZM2G077068_P01 | CC-NBS-LRR | 1 | 221,236,143 | 221,241,717 |
| 8 | ZmNBS63 | GRMZM2G045027_P01 | NBS-LRR | 1 | 225,341,943 | 225,345,923 |
| 9 | ZmNBS37 | GRMZM2G150179_P01 | CC-NBS-LRR | 1 | 232,526,764 | 232,530,976 |
| 10 | ZmNBS100 | GRMZM2G032602_P01 | NBS-LRR | 2 | 28,929,571 | 28,933,965 |
| 11 | ZmNBS99 | GRMZM2G450496_P01 | NBS-LRR | 2 | 97,109,835 | 97,124,968 |
| 12 | ZmNBS30 | GRMZM2G076474_P01 | CC-NBS-LRR | 2 | 113,360,426 | 113,363,678 |
| 13 | ZmNBS27 | GRMZM2G065692_P01 | CC-NBS-LRR | 2 | 134,037,600 | 134,048,302 |
| 14 | ZmNBS8 | GRMZM2G003755_P01 | CC-NBS-LRR | 2 | 134,132,718 | 134,138,847 |
| 15 | ZmNBS32 | GRMZM2G079082_P01 | CC-NBS-LRR | 2 | 209,124,511 | 209,128,692 |
| 16 | ZmNBS103 | GRMZM2G379770_P01 | NBS-LRR | 2 | 214,204,840 | 214,207,788 |
| 17 | ZmNBS56 | GRMZM2G449669_P01 | CC-NBS-LRR | 2 | 218,405,606 | 218,408,442 |
| 18 | ZmNBS29 | GRMZM2G074496_P01 | CC-NBS-LRR | 2 | 225,227,431 | 225,230,275 |
| 19 | ZmNBS91 | GRMZM2G145626_P01 | NBS-LRR | 2 | 225,987,147 | 225,990,695 |
| 20 | ZmNBS98 | GRMZM2G444543_P01 | NBS | 2 | 232,514,252 | 232,518,042 |
| 21 | ZmNBS79 | GRMZM2G007898_P01 | NBS-LRR | 2 | 234,264,294 | 234,268,222 |
| 22 | ZmNBS93 | GRMZM2G309503_P01 | NBS-LRR | 2 | 234,288,804 | 234,293,815 |
| 23 | ZmNBS87 | GRMZM2G094664_P01 | NBS-LRR | 2 | 236,717,181 | 236,721,015 |
| 24 | ZmNBS96 | GRMZM2G396357_P01 | NBS-LRR | 2 | 236,741,597 | 236,746,608 |
| 25 | ZmNBS85 | GRMZM2G091088_P01 | NBS-LRR | 3 | 61,339,824 | 61,342,588 |
| 26 | ZmNBS21 | GRMZM2G044724_P02 | CC-NBS | 3 | 85,659,561 | 85,663,470 |
| 27 | ZmNBS22 | GRMZM2G044724_P03 | CC-NBS-LRR | 3 | 85,659,561 | 85,663,470 |
| 28 | ZmNBS23 | GRMZM2G044724_P04 | CC-NBS-LRR | 3 | 85,659,561 | 85,663,341 |
| 29 | ZmNBS62 | GRMZM2G044724_P01 | CC-NBS-LRR | 3 | 85,659,561 | 85,663,504 |
| 30 | ZmNBS55 | AC230011.2_FGP002 | CC-NBS-LRR | 3 | 113,894,628 | 113,902,317 |
| 31 | ZmNBS82 | GRMZM2G064015_P01 | NBS | 3 | 132,383,623 | 132,385,474 |
| 32 | ZmNBS44 | GRMZM2G302279_P01 | CC-NBS | 3 | 132,610,360 | 132,612,175 |
| 33 | ZmNBS80 | GRMZM2G047652_P01 | NBS | 3 | 188,374,974 | 188,376,493 |
| 34 | ZmNBS41 | GRMZM2G178704_P01 | CC-NBS | 3 | 188,513,304 | 188,514,833 |
| 35 | ZmNBS69 | GRMZM2G173647_P01 | CC-NBS-LRR | 3 | 189,774,217 | 189,777,798 |
| 36 | ZmNBS34 | GRMZM2G116271_P01 | CC-NBS | 3 | 202,254,193 | 202,256,934 |
| 37 | ZmNBS57 | GRMZM2G455909_P01 | CC-NBS-LRR | 4 | 1,549,008 | 1,551,816 |
| 38 | ZmNBS33 | GRMZM2G087974_P01 | CC-NBS | 4 | 1,970,861 | 1,972,828 |
| 39 | ZmNBS46 | GRMZM2G333659_P01 | NBS | 4 | 15,603,027 | 15,613,201 |
| 40 | ZmNBS92 | GRMZM2G304049_P01 | NBS-LRR | 4 | 42,260,480 | 42,262,502 |
| 41 | ZmNBS25 | GRMZM2G050959_P01 | CC-NBS-LRR | 4 | 189,294,131 | 189,297,207 |
| 42 | ZmNBS94 | GRMZM2G327659_P01 | NBS-LRR | 4 | 200,734,715 | 200,738,675 |
| 43 | ZmNBS12 | GRMZM2G005347_P01 | CC-NBS-LRR | 4 | 203,229,838 | 203,233,710 |
| 44 | ZmNBS13 | GRMZM2G005347_P02 | CC-NBS-LRR | 4 | 203,233,710 | 203,234,037 |
| 45 | ZmNBS14 | GRMZM2G005452_P01 | CC-NBS-LRR | 4 | 203,279,153 | 203,285,455 |
| 46 | ZmNBS15 | GRMZM2G005452_P02 | CC-NBS-LRR | 4 | 203,279,428 | 203,282,559 |
| 47 | ZmNBS45 | GRMZM2G308064_P01 | CC-NBS-LRR | 4 | 203,459,531 | 203,469,250 |
| 48 | ZmNBS78 | GRMZM2G006780_P01 | NBS-LRR | 4 | 203,535,291 | 203,537,721 |
| 49 | ZmNBS64 | GRMZM2G051502_P01 | CC-NBS-LRR | 4 | 213,768,412 | 213,777,351 |
| 50 | ZmNBS83 | GRMZM2G454334_P01 | NBS-LRR | 5 | 31,421,323 | 31,423,336 |
| 51 | ZmNBS68 | GRMZM2G162098_P01 | CC-NBS-LRR | 5 | 55,431,018 | 55,435,275 |
| 52 | ZmNBS65 | GRMZM2G091672_P01 | CC-NBS-LRR | 5 | 55,549,877 | 55,553,874 |
| 53 | ZmNBS86 | GRMZM2G091696_P01 | NBS-LRR | 5 | 55,562,326 | 55,571,461 |
| 54 | ZmNBS66 | GRMZM2G105428_P01 | CC-NBS-LRR | 5 | 55,781,394 | 55,784,962 |
| 55 | ZmNBS89 | GRMZM2G454718_P01 | NBS | 5 | 55,949,527 | 55,951,065 |
| 56 | ZmNBS72 | GRMZM2G469414_P01 | CC-NBS-LRR | 5 | 57,205,927 | 57,212,295 |
| 57 | ZmNBS101 | GRMZM2G060583_P01 | NBS-LRR | 5 | 64,774,836 | 64,779,944 |
| 58 | ZmNBS51 | GRMZM2G385979_P02 | CC-NBS-LRR | 5 | 204,042,649 | 204,048,611 |
| 59 | ZmNBS70 | GRMZM2G385979_P01 | CC-NBS-LRR | 5 | 204,042,649 | 204,046,394 |
| 60 | ZmNBS81 | GRMZM2G054946_P01 | CC-NBS-LRR | 6 | 5,441,476 | 5,444,796 |
| 61 | ZmNBS75 | AC193598.3_FGP002 | NBS-LRR | 6 | 5,503,430 | 5,507,323 |
| 62 | ZmNBS47 | GRMZM2G334584_P01 | CC-NBS | 6 | 8,097,363 | 8,102,256 |
| 63 | ZmNBS4 | AC195587_FGP004 | CC-NBS-LRR | 6 | 78,610,403 | 78,619,923 |
| 64 | ZmNBS7 | GRMZM2G002656_P01 | CC-NBS-LRR | 6 | 128,823,409 | 128,833,681 |
| 65 | ZmNBS35 | GRMZM2G116335_P01 | CC-NBS | 6 | 155,597,135 | 155,598,686 |
| 66 | ZmNBS105 | GRMZM2G382273_P01 | NBS-LRR | 7 | 28,265,804 | 28,270,839 |
| 67 | ZmNBS20 | GRMZM2G038388_P01 | CC-NBS-LRR | 7 | 28,319,273 | 28,323,150 |
| 68 | ZmNBS104 | GRMZM2G381802_P01 | NBS-LRR | 7 | 78,982,082 | 78,988,141 |
| 69 | ZmNBS40 | GRMZM2G176403_P01 | CC-NBS-LRR | 7 | 140,258,657 | 140,259,754 |
| 70 | ZmNBS52 | GRMZM2G397557_P01 | CC-NBS-LRR | 7 | 148,700,092 | 148,704,194 |
| 71 | ZmNBS18 | GRMZM2G028713_P01 | CC-NBS-LRR | 7 | 148,734,857 | 148,747,797 |
| 72 | ZmNBS60 | GRMZM2G017603_P01 | CC-NBS-LRR | 8 | 104,379,532 | 104,382,273 |
| 73 | ZmNBS24 | GRMZM2G047152_P01 | CC-NBS-LRR | 8 | 104,460,886 | 104,464,505 |
| 74 | ZmNBS49 | GRMZM2G351921_P01 | CC-NBS-LRR | 8 | 131,531,703 | 131,540,072 |
| 75 | ZmNBS39 | GRMZM2G169584_P01 | CC-NBS | 8 | 155,541,108 | 155,543,379 |
| 76 | ZmNBS38 | GRMZM2G169571_P01 | CC-NBS | 8 | 155,581,018 | 155,582,701 |
| 77 | ZmNBS16 | GRMZM2G016802_P01 | CC-NBS-LRR | 8 | 160,531,062 | 160,534,322 |
| 78 | ZmNBS17 | GRMZM2G016802_P02 | CC-NBS-LRR | 8 | 160,531,062 | 160,534,220 |
| 79 | ZmNBS53 | GRMZM2G403151_P01 | CC-NBS-LRR | 9 | 19,231,482 | 19,238,473 |
| 80 | ZmNBS42 | GRMZM2G180244_P01 | CC-NBS-LRR | 10 | 2,049,440 | 2,055,121 |
| 81 | ZmNBS43 | GRMZM2G180254_P01 | CC-NBS-LRR | 10 | 2,124,313 | 2,130,521 |
| 82 | ZmNBS54 | GRMZM2G437314_P01 | NBS-LRR | 10 | 2,257,163 | 2,260,980 |
| 83 | ZmNBS6 | AC234175.1_FGP009 | CC-NBS-LRR | 10 | 2,322,125 | 2,362,787 |
| 84 | ZmNBS9 | GRMZM2G004412_P01 | CC-NBS-LRR | 10 | 2,984,369 | 2,993,434 |
| 85 | ZmNBS3 | AC152495.1_FGP002 | CC-NBS-LRR | 10 | 3280568 | 3,284,464 |
| 86 | ZmNBS73 | AC152495.1_FGP003 | NBS | 10 | 3,297,020 | 3,304,271 |
| 87 | ZmNBS2 | AC152495.1_FGP010 | CC-NBS-LRR | 10 | 3,369,199 | 3,373,083 |
| 88 | ZmNBS74 | AC152495.1_FGP015 | NBS-LRR | 10 | 3,401,150 | 3,402,412 |
| 89 | ZmNBS1 | AC152495.1_FGP017 | NBS-LRR | 10 | 3,438,490 | 3,441,711 |
| 90 | ZmNBS28 | GRMZM2G069382_P01 | CC-NBS | 10 | 3,569,035 | 3,587,751 |
| 91 | ZmNBS84 | GRMZM2G083258_P01 | NBS | 10 | 3,646,298 | 3,647,374 |
| 92 | ZmNBS90 | GRMZM2G143769_P01 | NBS-LRR | 10 | 3,684,166 | 3,685,815 |
| 93 | ZmNBS59 | GRMZM2G443939_P02 | CC-NBS-LRR | 10 | 3,698,044 | 3,702,576 |
| 94 | ZmNBS76 | GRMZM2G003625_P01 | NBS | 10 | 3,846,478 | 3,851,179 |
| 95 | ZmNBS77 | GRMZM2G003625_P02 | NBS | 10 | 3,846,864 | 3,851,026 |
| 96 | ZmNBS26 | GRMZM2G061742_P01 | CC-NBS-LRR | 10 | 3,904,402 | 3,908,700 |
| 97 | ZmNBS10 | GRMZM2G005134_P01 | CC-NBS-LRR | 10 | 3,980,743 | 3,986,963 |
| 98 | ZmNBS11 | GRMZM2G005134_P02 | CC-NBS-LRR | 10 | 3,980,743 | 3,986,963 |
| 99 | ZmNBS48 | GRMZM2G350841_P01 | CC-NBS-LRR | 10 | 5,003,131 | 5,007,545 |
| 100 | ZmNBS95 | GRMZM2G381429_P01 | NBS-LRR | 10 | 9,903,545 | 9,916,726 |
| 101 | ZmNBS5 | AC203972_FGP001 | CC-NBS-LRR | 10 | 27,647,980 | 27,650,691 |
| 102 | ZmNBS19 | GRMZM2G032751_P01 | CC-NBS-LRR | 10 | 69,601,329 | 69,607,535 |
| 103 | ZmNBS36 | GRMZM2G142680_P01 | CC-NBS-LRR | 10 | 94,965,435 | 94,969,104 |
| 104 | ZmNBS88 | GRMZM2G098677_P01 | NBS-LRR | 10 | 97,315,266 | 97,319,525 |
| 105 | ZmNBS97 | GRMZM2G397785_P01 | NBS-LRR | 10 | 97,363,160 | 97,369,329 |
| 106 | ZmNBS106 | GRMZM2G397788_P01 | NBS-LRR | 10 | 97,372,330 | 97,374,758 |
| 107 | ZmNBS50 | GRMZM2G353185_P01 | CC-NBS | 10 | 117,815,213 | 117,821,121 |

Note: The map position refers to the most likely position on the IBM2 neighbors 2008 map
