# Supplementary material for: Evidence of Multiple Disease Resistance (MDR) and Implication of Meta-Analysis in Marker Assisted Selection
Source: PLoS One. 2013 Jul 10;8(7):e68150. doi: 10.1371/journal.pone.0068150 (PMC3707948; doi:10.1371/journal.pone.0068150)
Supplement: Table S2 — Description of all the QTL observed during the meta-analysis and the number of real QTL. (DOC) [file pone.0068150.s007.doc]

Table S2 Description of all the QTL observed during the meta-analysis and the number of real QTL

| No | Chr | P. Pos | CI | L. Pos | R. Pos | L. Marker | R. Marker | Num* | Type^#^ |
| --- | --- | --- | --- | --- | --- | --- | --- | --- | --- |
| 1 | 1 | 89.09 | 7.1 | 85.6 | 92.6 | gst12 | IDP7711 | 7 | NS |
| 2 | 1 | 257.86 | 3.3 | 256.2 | 259.5 | IDP142 | IDP6818 | 4 | NS |
| 3 | 1 | 415.54 | 3.0 | 414.0 | 417.1 | TIDP7103 | pza03240 | 7 | NSG |
| 4 | 1 | 461.12 | 12.8 | 454.7 | 467.5 | TIDP5752 | AY112092 | 7 | NSG |
| 5 | 1 | 518.26 | 2.5 | 517.0 | 519.5 | IDP1951 | umc1590 | 4 | NS |
| 6 | 1 | 614.32 | 5.8 | 611.4 | 617.2 | IDP8161 | gpm820f | 7 | NS |
| 7 | 1 | 781.53 | 8.3 | 777.4 | 785.7 | gpm243b | umc1446 | 5 | NSG |
| 8 | 1 | 917.13 | 10.1 | 912.1 | 922.2 | bnlg1720 | npi407 | 4 | NS |
| 9 | 1 | 1006.14 | 4.7 | 1003.8 | 1008.5 | ao3 | gdh1 | 3 | NS |
| 10 | 2 | 51.03 | 1.9 | 50.1 | 52.0 | TIDP6110 | bnlg1338 | 7 | N |
| 11 | 2 | 151.75 | 2.8 | 150.3 | 153.2 | TIDP3392 | IDP225 | 10 | NS |
| 12 | 2 | 214.63 | 8.2 | 210.6 | 218.7 | agrr113a | rws1 | 6 | NSG |
| 13 | 2 | 274.45 | 5.0 | 272.0 | 277.0 | AY107218 | ras1 | 1 | N |
| 14 | 2 | 307.32 | 5.5 | 304.6 | 310.1 | mmp91 | IDP200 | 4 | NSG |
| 15 | 2 | 340.52 | 5.3 | 337.9 | 343.2 | phm3457 | IDP8654 | 1 | S |
| 16 | 2 | 373.88 | 3.4 | 372.2 | 375.6 | pza02939 | IDP3911 | 4 | NS |
| 17 | 2 | 451.77 | 5.6 | 449.0 | 454.6 | gpm178a | phm3668 | 4 | SG |
| 18 | 2 | 451.77 | 13.2 | 445.2 | 458.4 | magi50422 | IDP309 | 4 | SG |
| 19 | 2 | 645.23 | 2.4 | 644.0 | 646.5 | pza02727 | gpm717a | 5 | SG |
| 20 | 3 | 62.86 | 19.8 | 53.0 | 72.7 | umc2377 | TIDP4674 | 3 | NS |
| 21 | 3 | 166.06 | 2.9 | 164.6 | 167.5 | IDP697 | umc2000 | 11 | S |
| 22 | 3 | 229.53 | 0.9 | 229.1 | 231.5 | cef1 | IDP2431 | 6 | S |
| 23 | 3 | 263.12 | 18.9 | 253.7 | 272.5 | AY111333 | umc1750 | 4 | NS |
| 24 | 3 | 334.8 | 3.1 | 333.3 | 336.3 | AY111507 | cko2 | 5 | NSG |
| 25 | 3 | 384.67 | 4.8 | 382.3 | 387.1 | AY106230 | umc1400 | 5 | NG |
| 26 | 3 | 522.55 | 6.1 | 519.5 | 525.6 | dupssr17 | phm13673 | 7 | NSG |
| 27 | 3 | 647.15 | 6.1 | 644.1 | 650.2 | BE639338 | BE639338 | 4 | NS |
| 28 | 3 | 729.99 | 5.3 | 727.4 | 732.6 | IDP351 | pco135758 | 1 | N |
| 29 | 3 | 777.14 | 2.4 | 776.0 | 778.3 | pza00402 | IDP398 | 4 | NS |
| 30 | 4 | 105.18 | 13.5 | 98.4 | 111.9 | IDP4086 | IDP4153 | 3 | NS |
| 31 | 4 | 225.56 | 6.6 | 222.3 | 228.9 | IDP4938 | umc1652 | 2 | SG |
| 32 | 4 | 281.12 | 4.7 | 278.8 | 283.5 | pza00445 | nnr1 | 7 | NSG |
| 33 | 4 | 360.21 | 13.1 | 353.7 | 366.8 | umc2027 | AY110310 | 2 | N |
| 34 | 4 | 441.73 | 10.4 | 436.5 | 446.9 | IDP7898 | IDP7899 | 3 | NS |
| 35 | 4 | 451.04 | 3.2 | 449.4 | 452.7 | rpl29 | asg85a | 6 | NS |
| 36 | 4 | 479.46 | 45.8 | 456.6 | 502.4 | cl32627_1e | ufg23 | 2 | NS |
| 37 | 4 | 517.02 | 2.5 | 515.8 | 518.3 | AY110631 | npi270 | 2 | SG |
| 38 | 4 | 573.82 | 1.9 | 572.9 | 574.8 | IDP7752 | pza00155 | 3 | NS |
| 39 | 4 | 619.91 | 6.6 | 616.6 | 623.2 | CL2227_3 | umc2360 | 3 | S |
| 40 | 5 | 26.8 | 16.7 | 18.5 | 35.2 | AY109758 | PCO062666 | 1 | S |
| 41 | 5 | 99.6 | 7.8 | 95.7 | 103.5 | AY106121 | gpm610b | 3 | NS |
| 42 | 5 | 231.8 | 27.0 | 218.3 | 245.3 | umc27a | grp3 | 3 | NG |
| 43 | 5 | 304.06 | 2.5 | 302.8 | 305.3 | umn388 | gpm808 | 8 | NSG |
| 44 | 5 | 312.03 | 2.6 | 310.7 | 313.3 | umc1629 | bnlg1287 | 6 | NSG |
| 45 | 5 | 338.87 | 33.8 | 322.0 | 355.8 | umc1747 | ae1 | 6 | NSG |
| 46 | 5 | 399.2 | 9.9 | 394.2 | 404.2 | TIDP6514 | mmp47 | 5 | NG |
| 47 | 5 | 470.9 | 6.4 | 467.7 | 474.1 | IDP237 | CG738019 | 8 | NG |
| 48 | 5 | 487.35 | 9.5 | 482.6 | 492.1 | umc14c | umc1752 | 4 | NS |
| 49 | 5 | 621 | 30.1 | 606.0 | 636.0 | IDP181a | npi288a | 1 | S |
| 50 | 6 | 60.93 | 9.5 | 56.2 | 65.7 | umc1883 | AY110100 | 2 | S |
| 51 | 6 | 121.54 | 8.3 | 117.4 | 125.7 | mmp4 | umc1006 | 4 | S |
| 52 | 6 | 277.68 | 9.6 | 272.9 | 282.5 | umc1352a | csu360 | 3 | NS |
| 53 | 6 | 316.99 | 6.5 | 313.7 | 320.2 | umc46 | gpm709b | 3 | NS |
| 54 | 6 | 336.32 | 7.0 | 332.8 | 339.8 | IDP2375 | IDP8070 | 2 | NS |
| 55 | 6 | 346.5 | 3.3 | 344.9 | 348.2 | IDP7501 | pza02472 | 1 | N |
| 56 | 6 | 370.7 | 9.9 | 365.8 | 375.7 | uaz121a | IDP7652 | 2 | S |
| 57 | 6 | 489.19 | 14.3 | 482.0 | 496.3 | npi419a | mmp113 | 2 | NS |
| 58 | 7 | 182.7 | 12.0 | 176.7 | 188.7 | gbss1b | pza01607 | 3 | G |
| 59 | 7 | 220.08 | 2.7 | 218.8 | 221.4 | IDP8366 | gpm505 | 6 | NSG |
| 60 | 7 | 289.96 | 20.9 | 279.5 | 300.4 | IDP6980 | umc1713 | 6 | SG |
| 61 | 7 | 327.84 | 12.9 | 321.4 | 334.3 | mmp46 | zmm7 | 4 | N |
| 62 | 7 | 459.18 | 15.0 | 451.7 | 466.7 | IDP7252 | bcd349 | 1 | N |
| 63 | 7 | 535.87 | 109.4 | 481.2 | 590.6 | TIDP5805 | umc2197 | 1 | N |
| 64 | 8 | 130.06 | 12.0 | 124.1 | 136.1 | lrk1 | bcd1823b | 2 | NS |
| 65 | 8 | 171.79 | 11.1 | 166.2 | 177.4 | gpm178b | umc124a | 3 | S |
| 66 | 8 | 204.7 | 8.9 | 200.3 | 209.1 | umc1868 | umc1802 | 3 | S |
| 67 | 8 | 333.73 | 2.4 | 332.5 | 334.9 | sdg105a | cyc1 | 5 | NS |
| 68 | 8 | 367.88 | 2.7 | 366.5 | 369.2 | IDP7284 | gpm870b | 4 | NS |
| 69 | 8 | 377.34 | 4.4 | 375.2 | 379.5 | umc2401 | hda103 | 7 | NSG |
| 70 | 8 | 437.75 | 35.0 | 420.3 | 455.3 | AY107140 | IDP5847 | 3 | NSG |
| 71 | 8 | 481.51 | 36.2 | 463.4 | 499.6 | IDPnpi268 | gpm204a | 1 | N |
| 72 | 8 | 541.14 | 6.9 | 537.7 | 544.6 | gpm685 | umc1933 | 3 | NSG |
| 73 | 8 | 552.86 | 20.0 | 542.9 | 562.8 | umc1933 | IDP848 | 2 | NG |
| 74 | 9 | 84.83 | 9.3 | 80.2 | 89.5 | IDP1969 | TIDP3336 | 2 | NS |
| 75 | 9 | 109.97 | 11.8 | 104.1 | 115.9 | chr113 | bnlg1372 | 4 | NS |
| 76 | 9 | 142.84 | 9.4 | 138.1 | 147.5 | hyp1 | hvp1 | 3 | NS |
| 77 | 9 | 177.83 | 29.9 | 162.9 | 192.8 | pco067521 | TIDP5788 | 3 | NS |
| 78 | 9 | 236.66 | 1.3 | 236.0 | 237.3 | TIDP6547 | TIDP6547 | 3 | NS |
| 79 | 9 | 245.22 | 8.7 | 240.9 | 249.6 | IDP8040 | std6a | 5 | NSG |
| 80 | 9 | 294.16 | 9.7 | 289.3 | 299.0 | gpm292a | expb1 | 4 | NS |
| 81 | 9 | 329.55 | 1.6 | 328.7 | 330.4 | pco109929 | ufg64 | 5 | SG |
| 82 | 10 | 173.85 | 13.2 | 167.3 | 180.4 | IDP8390 | pza02961 | 3 | NS |
| 83 | 10 | 228.43 | 14.0 | 221.4 | 235.4 | gpm554 | csu969b | 3 | NS |
| 84 | 10 | 245.22 | 2.7 | 243.9 | 246.6 | cl37957_1 | umc1995 | 1 | S |
| 85 | 10 | 280.5 | 24.4 | 268.3 | 292.7 | TIDP5547 | php15013 | 3 | S |
| 86 | 10 | 339.01 | 7.6 | 335.2 | 342.8 | gpm714b | IDPrf2ejr1 | 6 | NSG |
| 87 | 10 | 378.57 | 17.3 | 369.9 | 387.2 | pco101731 | gpm31b | 2 | S |
| 88 | 10 | 447.89 | 6.5 | 444.6 | 451.1 | TIDP5143 | sgb103 | 1 | S |

Note: Chr (Chromosome number), P. Pos (Peak position of the QTL), CI (Confidence interval), L. Pos (Left possible position), R. Pos (Right possible position), L. Marker (Left possible flanking marker), R. Marker (Right possible flanking marker) according to consensus IBM2 map 2008. * is the number of QTL and ^#^ in the type of QTL for the three diseases (N stands for NLB, S for SLB and G for GLS).
