# Supplementary material for: Evidence of Multiple Disease Resistance (MDR) and Implication of Meta-Analysis in Marker Assisted Selection
Source: PLoS One. 2013 Jul 10;8(7):e68150. doi: 10.1371/journal.pone.0068150 (PMC3707948; doi:10.1371/journal.pone.0068150)
Supplement: Table S3 — Identified NBS-encoding genes, Ht1, and Ht2 genes in the meta-analysis along with the physical position on different chromosomes responsible for disease resistance. (DOC) [file pone.0068150.s008.doc]

Table S3 Identified NBS-encoding genes, *Ht1*, and *Ht2* genes in the meta-analysis along with the physical position on different chromosomes responsible for disease resistance

| No | Chr | L. physical | R. physical | NBS Pos | Gene | Gene ID |
| --- | --- | --- | --- | --- | --- | --- |
| 1 | 1 | 7996167 | 9379775 |  |  |  |
| 2 | 1 | 41277176 | 43133756 |  |  |  |
| 3 | 1 | 87363085 | 92337988 |  |  |  |
| 4 | 1 | 147463300 | 172065774 | 164,086,816 | CC-NBS-LRR | GRMZM2G443525_P01 |
|  |  |  |  | 164,089,066 | NBS-LRR | GRMZM2G443525_P02 |
| 5 | 1 | 181193652 | 183808551 |  |  |  |
| 6 | 1 | 199274772 | 200547561 |  |  |  |
| 7 | 1 | 236141034 | 240784199 |  |  |  |
| 8 | 1 | 274684822 | 275484407 |  |  |  |
| 9 | 1 | 283187143 | 287296815 |  |  |  |
| 10 | 2 | 4180210 | 4665846 |  |  |  |
| 11 | 2 | 13382002 | 13827670 |  |  |  |
| 12 | 2 | 19294899 | 21942122 |  |  | *Ht1* |
| 13 | 2 | 34149431 | 38698258 |  |  |  |
| 14 | 2 | 45986220 | 49928821 |  |  |  |
| 15 | 2 | 61801414 | 64657292 |  |  |  |
| 16 | 2 | 152207394 | 163319683 |  |  |  |
| 17 | 2 | 196055747 | 198783076 |  |  |  |
| 18 | 2 | 196219045 | 199166751 |  |  |  |
| 19 | 2 | 230191138 | 232639640 | 232,514,252 | NBS | GRMZM2G444543_P01 |
| 20 | 3 | 3478666 | 4845444 |  |  |  |
| 21 | 3 | 14790768 | 17287483 |  |  |  |
| 22 | 3 | 49139829 | 56820117 |  |  |  |
| 23 | 3 | 95264931 | 118147511 | 113,894,628 | CC-NBS-LRR | AC230011.2_FGP002 |
| 24 | 3 | 148555854 | 152399151 |  |  |  |
| 25 | 3 | 162239008 | 166460886 |  |  |  |
| 26 | 3 | 190889172 | 196152996 |  |  |  |
| 27 | 3 | 213499670 | 213644376 |  |  |  |
| 28 | 3 | 218016295 | 219663768 |  |  |  |
| 29 | 3 | 223154500 | 223672629 |  |  |  |
| 30 | 4 | 5368369 | 6546782 |  |  |  |
| 31 | 4 | 23257842 | 26437539 |  |  |  |
| 32 | 4 | 46450572 | 56313225 |  |  |  |
| 33 | 4 | 153691967 | 157609682 |  |  |  |
| 34 | 4 | 177339850 | 179795723 |  |  |  |
| 35 | 4 | 179742246 | 181394039 |  |  |  |
| 36 | 4 | 181199717 | 186679900 |  |  |  |
| 37 | 4 | 188203557 | 190107550 |  |  |  |
| 38 | 4 | 213780571 | 214143007 |  |  |  |
| 39 | 4 | 231469494 | 234515102 |  |  |  |
| 40 | 5 | 0 | 2209545 |  |  |  |
| 41 | 5 | 5218115 | 6178263 |  |  |  |
| 42 | 5 | 19559066 | 31445711 |  |  |  |
| 43 | 5 | 78362776 | 84252685 |  |  |  |
| 44 | 5 | 111377269 | 147622510 |  |  |  |
| 45 | 5 | 137387173 | 168468750 |  |  |  |
| 46 | 5 | 172391859 | 175787174 |  |  |  |
| 47 | 5 | 188931436 | 192549407 | 189,294,131 | CC-NBS-LRR | GRMZM2G050959_P01 |
| 48 | 5 | 194636117 | 195590488 |  |  |  |
| 49 | 5 | 205440973 | 217012402 | 213,768,412 | CC-NBS-LRR | GRMZM2G051502_P01 |
| 50 | 6 | 2776439 | 8680351 |  |  |  |
| 51 | 6 | 36557807 | 89004837 |  |  |  |
| 52 | 6 | 131974895 | 142486587 |  |  |  |
| 53 | 6 | 143461102 | 148018223 |  |  |  |
| 54 | 6 | 147223982 | 148254670 |  |  |  |
| 55 | 6 | 148827182 | 149676378 |  |  |  |
| 56 | 6 | 150891712 | 152030649 |  |  |  |
| 57 | 6 | 162662563 | 163917221 |  |  |  |
| 58 | 7 | 28146857 | 83126496 | 78,610,403 | CC-NBS-LRR | AC195587_FGP004 |
| 59 | 7 | 92084656 | 100656705 |  |  |  |
| 60 | 7 | 127969663 | 129866479 | 128,823,409 | CC-NBS-LRR | GRMZM2G002656_P01 |
| 61 | 7 | 132596480 | 138763032 |  |  |  |
| 62 | 7 | 161803643 | 162171955 |  |  |  |
| 63 | 7 | 163209668 | 170998616 |  |  |  |
| 64 | 8 | 10,851,783 | 14124841 |  |  |  |
| 65 | 8 | 20151490 | 22380791 |  |  |  |
| 66 | 8 | 34593113 | 71005804 |  |  |  |
| 67 | 8 | 120063628 | 124952242 |  |  |  |
| 68 | 8 | 133561516 | 135103696 |  |  |  |
| 69 | 8 | 134862198 | 140298697 |  |  |  |
| 70 | 8 | 160297834 | 165223431 | 160,531,062 | *Ht2*  CC-NBS-LRR | GRMZM2G016802_P01 |
|  |  |  |  | 160,531,062 | CC-NBS-LRR | GRMZM2G016802_P02 |
| 71 | 8 | 165636122 | 169527442 |  |  |  |
| 72 | 8 | 171401538 | 171705679 |  |  |  |
| 73 | 8 | 171334858 | 173943272 |  |  |  |
| 74 | 9 | 11234873 | 12324396 |  |  |  |
| 75 | 9 | 12942672 | 15145277 |  |  |  |
| 76 | 9 | 15975038 | 18607113 |  |  |  |
| 77 | 9 | 16660671 | 22680841 | 19,231,482 | CC-NBS-LRR | GRMZM2G403151_P01 |
| 78 | 9 | 28505158 | 28507533 |  |  |  |
| 79 | 9 | 88828272 | 96247821 |  |  |  |
| 80 | 9 | 113013688 | 119479379 |  |  |  |
| 81 | 9 | 130485871 | 133595334 |  |  |  |
| 82 | 10 | 15798991 | 70685970 | 27,647,980 | CC-NBS-LRR | AC203972_FGP001 |
|  |  |  |  | 69,601,329 | CC-NBS-LRR | GRMZM2G032751_P01 |
| 83 | 10 | 83332319 | 87309153 |  |  |  |
| 84 | 10 | 88248022 | 92990601 |  |  |  |
| 85 | 10 | 112431185 | 122924003 | 117,815,213 | CC-NBS | GRMZM2G353185_P01 |
| 86 | 10 | 130953353 | 133313854 |  |  |  |
| 87 | 10 | 136333972 | 139101240 |  |  |  |
| 88 | 10 | 143391161 | 146296685 |  |  |  |
